# Association of Variants of 786T>C (rs2070744) Polymorphism of the *NOS3* Gene with Age-Dependent Electrophysiological Disorders in Myocardium

**DOI:** 10.17691/stm2025.17.5.06

**Published:** 2025-10-31

**Authors:** I.V. Averyanova, A.N. Loskutova, I.N. Bezmenova

**Affiliations:** DSc, Head of Laboratory; Chief Researcher, Laboratory of Physiology in Extreme Conditions; Scientific Research Centre “Arktika”, Far Eastern Branch of the Russian Academy of Sciences (SRC “Arktika” FЕВ RAS), 24 Karla Marxa Avenue, Magadan, 685000, Russia; PhD, Senior Researcher, Laboratory of Physiology in Extreme Conditions; Scientific Research Centre “Arktika”, Far Eastern Branch of the Russian Academy of Sciences (SRC “Arktika” FЕВ RAS), 24 Karla Marxa Avenue, Magadan, 685000, Russia; PhD, Researcher, Laboratory of Physiology in Extreme Conditions; Scientific Research Centre “Arktika”, Far Eastern Branch of the Russian Academy of Sciences (SRC “Arktika” FЕВ RAS), 24 Karla Marxa Avenue, Magadan, 685000, Russia

**Keywords:** 786T>C (rs2070744) polymorphism of the *NOS3* gene, ECG dispersion mapping, ontogenesis, age dynamics

## Abstract

**The aim of the study** was to examine associations of ECG signal micro-alternations values with various variants of the 786T>C (rs2070744) polymorphism of the *NOS3* gene in cohorts of mature and elderly male Northerners.

**Materials and Methods:**

One hundred forty male residents of the Magadan Region (Russia) aged 25–80 were included in the study. All the examinees were classified into mature and elderly. Men of different ages were divided into two groups based on the presence/ absence of the NOS3*C allele in their genotypes: group 1 consisted of homozygous men (TT genotype); group 2 — of heterozygous and homozygous carriers of the NOS3*C allele (TC+CC genotypes). Genetic SNP testing and methods for assessing the characteristics of cardiac variance mapping were used.

**Results:**

The study revealed age-associated impairment of dispersion characteristics of ECG signal, the “Myocardium” and “Rhythm” indices, as well as myocardial electrophysiological disorders, which were more characteristic of the elderly men who were carriers of the minor NOS3*C allele (genotypes TC+CC). This was accompanied by a synchronous increase in the “rigidity” of the matrix of ECG dispersion mapping indicators. The two-factor dispersion analysis showed that the 786T>C (rs2070744) polymorphism of the *NOS3* gene significantly influenced the ventricular hypertrophy (G9) at simultaneous cumulative impact with the age factor: the “Age” factor on the “Myocardium” index, right and left ventricular repolarization (G5, G6), and their depolarization symmetry (G7).

**Conclusion:**

The results indicate that monitoring studies of the main characteristics of ECG dispersion mapping parameters in addition to the evaluation of 786T>C (rs2070744) polymorphism of the *NOS3* gene must be given emphasis since it is crucial for the formation of personalized and preventive medicine. It has been established that the presence of the minor allele NOS3*C of the polymorphism 786T>C (rs2070744) of the *NOS3* locus can be considered as an additional risk factor for age-associated electrophysiological disorders of the myocardium.

## Introduction

At present, the method of electrocardiogram dispersion mapping (ECG DM) is widely used in clinical practice and population screening. Its advantages include early diagnosis and detection of changes in ECG signal micro-alternations. This method allows to assess ion-electrolyte and metabolic changes in various areas of the heart, manifested in deviations in myocardial de- and repolarization processes [1–3]. However, the current stage of development in biomedical sciences provides that the study of the physiological mechanisms of adaptive changes in the cardiovascular system requires consideration of the molecular genetic determinants influencing the development of hemodynamic abnormalities.

Nitric oxide (NO) is known as a pleiotropic molecule that plays a crucial role in maintaining cardiovascular equilibrium. NO has several important functions, including modulation of blood flow and circulation, regulation of thrombus formation, inflammation, and immune responses. NO is also involved in neural activity. Evidence of the NO influence on the regulation of cardiovascular functions was obtained [4]. Human body endogenously synthesizes NO from arginine by three isoforms of the nitric oxide synthase enzyme, encoded by different genes. Functional aspects of the heart largely depend on endothelial NO synthase (eNOS) [5], which is a product of the expression of the *NOS3* gene (chromosomal localization of the gene 7q35–7q36). Polymorphisms in the *NOS3* gene are closely associated with a higher incidence of cardiovascular diseases. For example, the C allele of the *NOS3* locus — a polymorphism 786T>C (rs2070744) — results in a decrease in eNOS expression and, thus, in a decrease in NO production, which in turn may be accompanied by an increased risk of hypertension [6], preeclampsia [7], diabetic nephropathy [8], and migraine [9]. Association of this allele with more frequent episodes of coronary vasospasm, aneurysm rupture followed by subarachnoid hemorrhage and vasospasm was proved [10, 11]. The *NOS3* gene is expressed not only in the vascular endothelium, but also in cardiomyocytes [12, 13], platelets [14], and erythrocytes [15].

In addition to molecular genetic determinants, environmental factors must also be considered. Extreme northern conditions are known to place stress on the cardiovascular system. This is due to the fact that prolonged exposure to cold leads to increased peripheral vascular tone and energy consumption [16]. A significant decrease in life expectancy was established for Arctic and northern regions compared to regions with favorable climatic conditions, as well as higher male mortality from circulatory diseases (up to 30% in some regions) [17, 18].

Thus, studies of molecular genetic determinants in the development of electrophysiological myocardial disorders in people living and working in the Arctic and subarctic climate zones are of particular relevance.

**The aim of the study** was to investigate the associations of ECG signal micro-alternations indices with various variants of the 786T>C (rs2070744) polymorphism of the *NOS3* gene in northern men of mature and elderly age.

## Materials and Methods

One hundred forty male residents of the Magadan Region aged 25–80 were included in the study. According to the age classification adopted at the International Symposium on Developmental Physiology (Moscow, Russia, 1965), two groups were formed: 75 mature (middle-aged) subjects (25–55 years old) and 65 elderly and old subjects (hereinafter referred to as the elderly) (61–80 years old).

The study was conducted in accordance with the Declaration of Helsinki and approved by the local Ethics Committee of the SRC “Arktika” FЕВ RAS (Russia) (Protocol No.002/021 dated November 26, 2021). Each patient submitted informed consent prior to inclusion in the study.

Blood from the cubital vein of volunteers, collected in 15 ml EDTA-preserved test tubes, served as the ***material for genetic testing***. Genomic DNA was isolated using phenol-chloroform. Genotyping of the 786T>C (rs 2070744) polymorphism of the *NOS3* gene was performed by polymerase chain reaction at the Human Molecular Genetics Laboratory of the Department of Medical and Biological Subjects of the Belgorod State National Research University (Russia) using commercial SNP-Screen kits (Synthol, Russia).

***The parameters of dispersion mapping of cardiac ECG*** were obtained using a KardioVizor-06s device (Medical Computer Systems, Russia). The myocardial micro-alternations index (“Myocardium”) was assessed: values less than 15% were considered normal; from 15 to 19% indicated borderline conditions (borderline values are at the edge between normal and pathological); values of 20% and above indicated potential pathologies (pathology). The “Rhythm” index (heart rate variability): absence of significant deviations corresponds to values less than 20%; values equal to 100% indicate the most pronounced changes in the characteristics of R–R intervals variability, which is typical of arrhythmias or severe stress. The severity and localization of electrophysiological changes in the de- and repolarization of the atrial and ventricular myocardium were reflected by the “Detailing code”, which included the time intervals of the PQRST cardiac complex: G1–G2 — depolarization of the right and left atria; G3–G4 — depolarization of the right (RV) and left (LV) ventricles; G5–G6 — depolarization of the RV and LV; G7 — symmetry of ventricular depolarization; G8 — intraventricular blocks; G9 — ventricular hypertrophy [19].

***Statistical data processing*** was performed using the Statistica 6.0 software. Genotype and allele frequencies were calculated in accordance with the Hardy–Weinberg law; genotype frequencies were compared using the χ^2^ criterion. The normality of the measured variables distribution was tested using the Shapiro–Wilk test. To characterize the results, the mean (M) and standard deviation (SD) were calculated. Intergroup differences were determined in each age group using the Student’s t-test for independent samples. Factor analysis using the principal component analysis with Varimax rotation and Kaiser normalization, as well as two-way analysis of variance (ANOVA), were used for multiple comparisons. Statistically significant differences between groups were determined by means of post-hoc analysis using the Scheffe multiple comparison test. The critical significance level was set at p≤0.05.

## Results and Discussion

The population distribution of genotype and allele frequencies for the studied locus was found to conform to Hardy–Weinberg equilibrium. SNP genotyping results indicate the two alleles in the sample: NOS3*T and NOS3*C.

The most common allele was NOS3*T, occurring at a frequency of 0.68 among the middle-aged men and at a frequency of 0.64 among the elderly men. The frequency of the minor allele NOS3*C, associated with physiologically reduced NO production, was 0.32 and 0.36 in middle-aged and elderly men, respectively.

For further analysis, each age group was divided into 2 subgroups by presence/absence of the NOS3*C allele in the genotype: group 1 were homozygous men (TT genotype), who had no minor NOS3*C allele and genetically retained normal production of physiological concentrations of NO (mature — 34 persons, elderly — 21 persons) whereas group 2 were heterozygous and homozygous carriers of the NOS3*C allele (TC+CC genotypes), who had genetically determined reduced production of NO by the vascular endothelium (mature — 41 persons, elderly — 44 persons).

The received data indicate that the values of the “Rhythm” index were associated with the age of the examined volunteers. For instance, in group 1, the index increased with age from 23±12 to 36±18%; p=0.003. Similar dynamics was observed in group 2 (TC+CC) — the index increased from 20±15 to 41±20%; p<0.001. The “Myocardium” index: in mature men, regardless of the manifestation of the NOS3*C allele in the genotype, average normal values generally prevailed (≥15%). In elderly men of group 1, the value of this index was 17±9%, which indicated insignificant changes in myocardial function to manifest borderline conditions, in elderly men of group 2 — 23±13%; p<0.01. Pathological manifestations among the elderly in group 2 (carriers of the minor allele, TC+CC genotypes) compared to mature men were due to RV and LV depolarization (G3–G4), LV repolarization (G6), and ventricular depolarization symmetry (G7); p<0.05.

**Figure 1.**
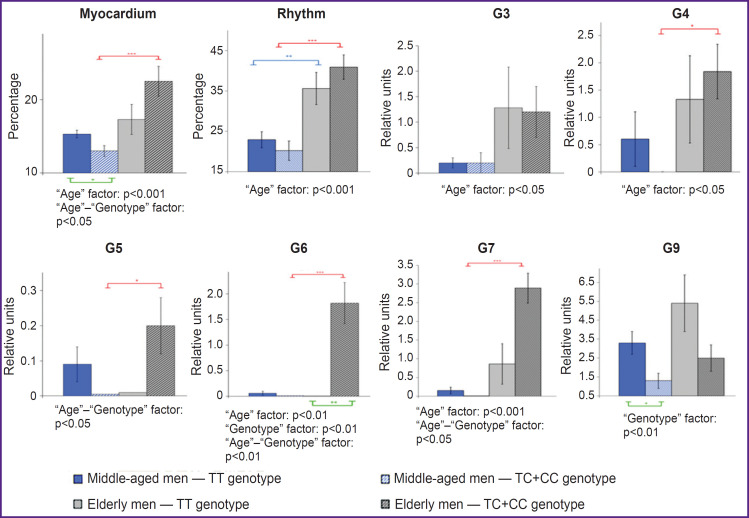
Electrocardiogram dispersion mapping parameters in groups of mature and elderly men with different genotypes for the 786T>C (rs2070744) polymorphism of the *NOS3* gene The results of a two-factor ANOVA analysis for comparing age-related parameters depending on genotype are presented under each graph: TT (*blue*), TC+CC (*red*) — according to the Scheffe test; intergroup differences (TT and TC+CC genotype) in each age group (*green*) — according to Student’s t-test for independent samples; * p<0.05; ** p<0.01; *** p<0.001. Myocardium — the “Myocardium” index, Rhythm — the “Rhythm” index, G — characteristics of the “Detailing code” (see “Materials and Methods”), HR — heart rate

Figure 1 demonstrates the most informative results of a two-factor analysis of variance (ANOVA) of ECG DM parameters in men with different genotypes for the 786T>C (rs2070744) polymorphism of the *NOS3* gene (G1–G2 and G8 were excluded, as they did not show statistically significant differences). It was established that the “Age” factor influenced the “Myocardium” (p=0.004) and “Rhythm” (p<0.001) indices, as well as micro-alternations in RV and LV depolarization (G3–G4; p=0.03), symmetry of ventricular de- and repolarization (G7) (p<0.001) and LV repolarization of the heart (G6) (p=0.005). At that, the combined mutual influence of both age and genetic (786T>C (rs2070744) polymorphism of the *NOS3* gene) factors was observed for the “Myocardium” index (p=0.02), as well as G5 (p=0.02), G6 (p=0.002), and G7 (p=0.04). A statistically significant influence of the genetic factor only (in Figure 1 — “Genotype”) was noted in relation to G6 (p=0.005) and G9 (p=0.007), which may indicate their age-associated and genetically determined influence on electrocardiogram dispersion mapping (ECG DM).

**Figure 2.**
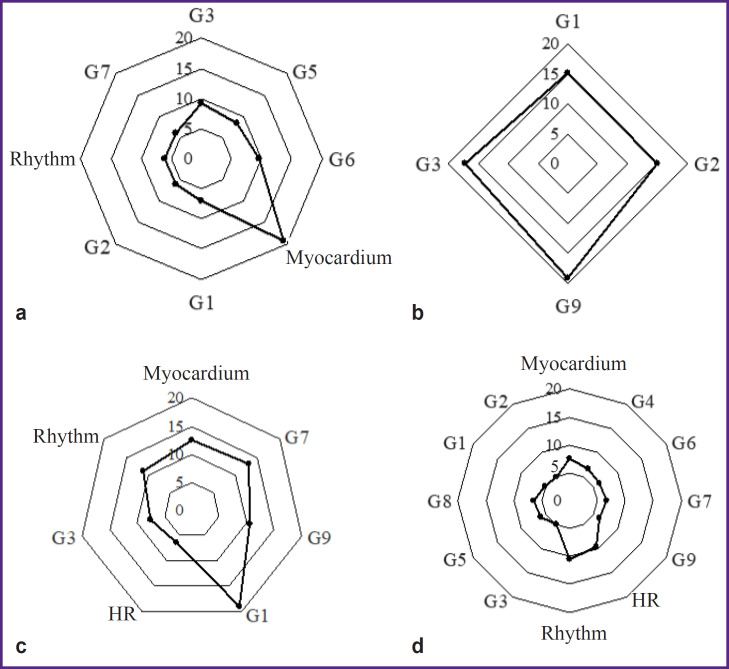
Matrices of one-factor analysis in the group of mature men with the TT genotype (a) and TC+CC (b), in elderly men with the TT genotype (c) and TC+CC (d) Myocardium — the “Myocardium” index, Rhythm — the “Rhythm” index, G — characteristics of the “Detailing code” (see “Materials and Methods”), HR — heart rate

Factor analysis was conducted to comprehensively evaluate the associations between electrocardiogram dispersion mapping parameters and the studied 786T>C (rs2070744) polymorphism of the *NOS3* gene in each age group. A quantitative estimate of the factor’s influence on ECG DM (as a percentage) was calculated, as well as the specific ECG value (as a percentage) of each parameter included in the factor structure (Tables 1 and 2). Based on the specific values, matrices of the main ECG DM parameters were prepared (Figure 2).

**T a b l e 1:** Results of the factor analysis in the group of mature and elderly men with the TT genotype

Mature age (n=34)				Elder age (n=21)			
Factor rank	Indicator	Indicator specific value in the factor structure (%)	Accumulative factor value (%)	Factor rank	Indicator	Indicator specific value in the factor structure (%)	Accumulative factor value (%)
1	G3	9.2	27	1	Myocardium	12.5	36
	G5	8.2			G7	13.0	
	G6	9.6			G9	10.5	
2	Myocardium	19.0	19	2	G1	19.0	19
3	G1	7.0	13	3	HR	6.4	14
	G2	6.0			G3	7.6	
4	Rhythm	6.0	12	4	Rhythm	11.0	11
	G7	6.0					
Total (%)			71	Total (%)			80

N o t e. Myocardium — the “Myocardium” index, Rhythm — the “Rhythm” index, G — characteristics of the “Detailing code” (see “Materials and Methods”), HR — heart rate.

**T a b l e 2:** Results of the factor analysis in the group of mature and elderly men with the ТС+СС genotype

Mature age (n=41)				Elder age (n=44)			
Factor rank	Indicator	Indicator specific value in the factor structure (%)	Accumulative factor value (%)	Factor rank	Indicator	Indicator specific value in the factor structure (%)	Accumulative factor value (%)
1	G1	15.1	30	1	Myocardium	7.6	32
	G2	14.9			G4	6.6	
					G6	6.1	
					G7	6.6	
					G9	6.1	
2	G9	19.0	19	2	Rhythm	10.5	20
					HR	9.5	
3	G3	17.0	17	3	G3	4.7	17
					G5	5.9	
					G8	6.4	
4	—			4	G1	5.2	10
					G2	4.8	
Total (%)			66	Total (%)			80

N o t e. Myocardium — the “Myocardium” index, “Rhythm” — the “Rhythm” index, G — characteristics of the “Detailing code” (see “Materials and Methods”), HR — heart rate.

In the sample of men in group 1 (TT homozygotes), minor changes in myocardial de- and repolarization were seen with age increasing, accompanied by a negative shift in heart rate regulation: an increase in the average values of the “Rhythm” index and an increase in the share of individuals with this index greater than 50%. A similar situation was seen in group 2 (TC+CC), subject to more pronounced deviations in myocardial de- and repolarization. These changes indicated deviations in heart rate variability from the normal condition and stress on the heart rhythm regulation systems [19]. The data are consistent with the results of the study conducted with participation of residents of the European North (Syktyvkar), which also reflected an increase in the “Rhythm” index with age [20]. Ivanov et al. [21] analyzed data of 537,820 persons from 25 regions of the Russian Federation and revealed practically zero dependence of the “Myocardium” index on age and sex. However, there is an age dependence on the prevalence of ranges of values of the “Myocardium” index: normal, borderline, and pathology. The study established a relationship between a decrease in the proportion of subjects with normal values of the “Myocardium” index and an increase in the prevalence of pathological and borderline values of the index from 55 and 35 years of age, respectively.

According to the data available to authors, individual cases with the “Myocardium” index greater than 20% were seen in mature men in the groups under consideration. In elderly men, the incidence increased to 6 and 13 cases in groups 1 and 2, respectively. Of 13 men in group 2 (TC+CC), a high probability of cardiac pathology (“Myocardium” index ≥50%) was diagnosed in 6 elderly men.

It should be noted that in group 2 of mature men (TC+CC), despite baseline “Myocardium” index values within the normal range, changes in micro-alternations during myocardial depolarization and repolarization were seen. The incidence of these abnormalities increased in elderly men: combined repolarization changes in the lateral and anterior LV (G6) were predominant. Moreover, G6 changes were significantly more common in elderly men compared to their peers in group 1 (TT).

The data in Figure 1 demonstrate statistically significant age-related dynamics of G4 and G7 only in men of group 2 (TC+CC), which was a sign of coronary blood flow disorders [1, 19]. Negative dynamics was also observed in relation to the dispersion fluctuations of the T wave (G5–G6), the increase of which is often associated with insufficient myocardial oxygenation. Here, the two- factor analysis of variance indicated the cumulative effect of age and genotype on these characteristics of the ECG DM. The individual set of manifestations of G1–G9 of the “Detailing code” should be taken into account, which can demonstrate different origins of deviations. Simultaneous deviations in G3–G4, G7 indicated pathological signs of myocardial ischemia, and changes in G7 with no deviations in G3–G4 indicated deviations of non-ischemic genesis [1, 19]. According to the data available to the authors, elderly men in group 1 (TT) with a predominance of borderline values for the said parameters had only one case of possible myocardial ischemia recorded. At that, elderly men in group 2 (TC+CC) demonstrated a combined incidence of borderline values of G3–G7 in five individuals, which clearly requires further clinical assessment.

The results of a two-factor analysis of variance (see Figure 1) indicated a statistically significant influence of the “Age” factor on micro-alternations in RV and LV depolarization (G3–G4), on the symmetry of ventricular depolarization (G7), and on LV repolarization (G6). Moreover, age has a complex effect on changes in the “Myocardium” and “Rhythm” indices. A joint statistically significant influence of the “Age” and “Genotype” factors was recorded for G5, G6, G7 and the “Myocardium” index, and the influence of the genetic factor only was noted for G6 and G9.

The results of the factor analysis showed that for mature men of group 1 (TT) of 12 ECG DM parameters included in the analysis 8 characteristics were combined into 4 factors with a total specific value of 71%. In elderly men of the same group, the matrix structure consisted of 4 factors and 7 parameters, with the total matrix value growth to 80% (see Table 1, Figure 2 (a), (c)).

In group 2 (TC+CC), in mature men, only 4 of 12 ECG DM parameters (3 factors) were included in the matrix structure with a total specific value of 66%. In the elder age, the set of factors determining the matrix increased to four, including all 12 analyzed ECG DM parameters with a total specific value of 80% (see Table 2, Figure 2 (b), (d)).

Currently, the “Myocardium” index is the main marker for the clinical interpretation of screening results [19]. The results of this study showed that in group 1 (no NOS3*C allele, TT homozygotes) in mature men, this index was represented in factor 2 with the highest specific value of 19%. In elderly men, it shifted to factor 1 with a value of 12.5% in the matrix structure. In men of group 2 (carriers of the minor NOS3*C allele, TC+CC genotypes), the “Myocardium” index appeared in the factor structure only for the elderly.

The G9 is considered the most sensitive indicator of compensatory and pathological responses of the ventricular myocardium [1, 19]. Thus, in mature men of group 1 (TT homozygotes), G9 did not make a significant contribution to accumulated analyzed indicators and was not included in the matrix structure. In elderly men, G9 was recorded in the structure of factor 1 with a specific value of 10.5%. For mature men of group 2 (TC+CC), G9 acted as an independent factor (factor 2) — 19%.

The provided matrices (see Figure 2) demonstrate that the key indicator in mature men of group 1 (TT) is the “Myocardium” index. In elderly men, the greatest contribution to the matrix was made by G1, reflecting all vegetative reactions, in particular the enhancement/ deficit of vagal influences on the heart rhythm [22].

A completely different picture was seen for men in group 2 (TC+CC). In mature men, the highest specific value in the matrix was attributed to G1–G2 (factor 1, 30%), which reflected the dispersion fluctuations of the P wave. This may indicate significant deviations in the body’s autonomic support even at this age. In elder age, the G1–G2 cluster moved to the last factor (factor 4), and the authors observed a transformation (unfolding) of the matrix: it included all 12 analyzed ECG DM parameters.

This change in the matrix structure, on the one side, demonstrated the ability of the cardiovascular system to maintain functioning under changing conditions (here, age-related conditions), but on the other side, it resulted in a decrease in the matrix plasticity. This can be considered the “price” the body pays for maintaining its vital functions under the influence of extreme environmental factors.

## Conclusion

These results indicate the need to conduct monitoring studies of the key ECG DM parameters in combination with the assessment of the *NOS3* gene polymorphism as a basis for developing personalized and preventive medicine. It was established that the NOS3*C minor allele of the 786T>C (rs2070744) polymorphism of the *NOS3* gene can be an additional risk factor for age- associated electrophysiological myocardial disorders in men living in extreme northern conditions.
